# Oral salt and water versus intravenous saline for the prevention of acute kidney injury following contrast-enhanced computed tomography: study protocol for a pilot randomized trial

**DOI:** 10.1186/s40697-015-0048-7

**Published:** 2015-04-16

**Authors:** Hiremath Swapnil, Greg A Knoll, Jeanne Françoise Kayibanda, Dean Fergusson, Benjamin JW Chow, Wael Shabana, Erin Murphy, Tim Ramsay, Matthew James, Christine A White, Amit Garg, Ron Wald, Jeffrey Hoch, Ayub Akbari

**Affiliations:** Division of Nephrology, Faculty of Medicine, University of Ottawa, Ottawa, Canada; Clinical Epidemiology Program, Ottawa Hospital Research Institute, Ottawa, Canada; Kidney Research Centre, Ottawa Health Research Institute, Ottawa, Canada; Division of Cardiology, University of Ottawa Heart Institute, Ottawa, Canada; Department of Medical Imaging, Faculty of Medicine, University of Ottawa, Ottawa, Canada; Ottawa Health Research Institute, Ottawa Hospital, Ottawa, Canada; Faculty of Medicine, Epidemiology& Community Medicine, University of Ottawa, Ottawa, Canada; Departments of Medicine and Community Health Sciences, University of Calgary, Alberta, Canada; Division of Nephrology, Department of Medicine, Queen’s University, Kingston, Canada; Division of Nephrology, Department of Medicine, University of Western Ontario, London, Canada; Division of Nephrology, Department of Medicine, St. Michael’s Hospital and University of Toronto, Toronto, Canada; Institute of Health Policy, Management and Evaluation, University of Toronto, Toronto, Canada; Division of Nephrology, The Ottawa Hospital, Riverside Campus, 1967 Riverside Drive, Ottawa, Ontario K1H 7 W9 Canada

**Keywords:** **C**ontrast-induced nephropathy, Oral Salt and Water, Intravenous saline, Randomized controlled trial

## Abstract

**Background:**

Although intravenous saline is the accepted prophylactic measure for the prevention of contrast- induced acute kidney injury, the oral route could offer an equivalent, practical, and cost saving approach. A systematic review of randomized trials that compared oral versus intravenous volume expansion for the prevention of radiocontrast-induced nephropathy in patients receiving arterial contrast reported no significant difference in the risk of contrast induced acute kidney injury between the oral and intravenous arms. Most trials for contrast nephropathy prevention have been in the setting of arterial contrast such as with cardiac catheterization, and not with venous contrast, such as computed tomography. The aim of this paper is to describe the protocol of a pilot trial comparing the effect of oral salt and water versus intravenous saline on the prevention of Acute Kidney Injury following contrast-enhanced computed tomography.

**Methods:**

Our study is a pilot, single-centre parallel randomized controlled trial. To be included, participants must be at stage 4 of chronic kidney disease as defined by a glomerular filtration rate <30 mL/min/1.73 m^2^, aged greater than 18 years and to undergo an outpatient contrast-enhanced computer tomography of the chest or abdomen. A total 50 patients will be randomised to receive either oral salt and water or intravenous isotonic saline. The primary outcome is feasibility, including estimates of recruitment rate, adherence to intervention and completeness of follow-up to assist in planning the definitive trial. The secondary outcome is safety and includes adverse events with oral salt and water loading as compared to intravenous isotonic saline.

**Discussion:**

The results of this pilot trial will provide critical information to plan a definitive trial to test the efficacy of the route of volume loading regimens in prevention of acute kidney injury after contrast-enhanced CT scans.

**Trial registration:**

The trial is registered at the US National Institutes of Health (ClinicalTrials.gov) # NCT02084771.

## Abrégé

**Contexte:**

L’administration prophylactique de solution saline par voie intraveineuse est utilisée pour la prévention de l’insuffisance rénale aiguë (IRA) secondaire aux produits de contraste iodés (PCI). Cependant, une administration par voie orale pourrait être un traitement alternatif équivalent, pratique et économique. Une revue systématique des essais cliniques à répartition aléatoire, qui comparaient les volémies post-administration orale, d’une part, et intraveineuse, d’autre part, de solution saline dans la prévention des néphropathies secondaires aux PCI de patients qui recevaient un agent de contraste par voie artérielle, a été effectuée. Aucun écart significatif en matière de risque d’IRA secondaire aux PCI n’a été observé entre les deux groupes à l’étude. La plupart des essais traitant de prévention des néphropathies secondaire aux PCI ont été effectués en contexte d’administration artérielle, comme par cathétérisme cardiaque, et non d’administration veineuse, comme par tomodensitométrie. Le but de cet article est de décrire le protocole d’un essai comparatif pilote des effets d’ une solution saline administrée par voie orale à ceux d'une solution saline isotonique administrée par voie intraveineuse dans la prévention de l’insuffisance rénale aiguë secondaire à la tomodensitométrie avec injection d’agent de contraste.

**Méthode:**

Notre étude est un essai comparatif pilote, unicentrique, à répartition aléatoire. Seuls les participants atteints de maladie rénale chronique de stade 4, définie par un débit de filtration glomérulaire < 30 ml/min/1,73m2, âgés de plus de 18 ans, et devant subir une tomodensitométrie avec agent de contraste du thorax ou de l’abdomen en clinique externe, pourront faire partie de l’échantillon. L’administration d’eau salée par voie orale ou de solution saline isotonique par voie intraveineuse sera répartie aléatoirement sur 50 patients. Les premières conclusions de l’étude aideront à planifier l’essai définitif. Celles-ci concernent la faisabilité, dont les estimations du taux de recrutement, d’adhésion à l'intervention et d’achèvement du suivi. Les conclusions secondaires se rapportent à la sécurité et font part, entre autres, d’une comparaison des effets indésirables de l’administration orale d’eau salée et de l’administration intraveineuse de solution saline isotonique.

**Discussion:**

Les résultats de cet essai pilote fourniront de l’information cruciale pour la planification de l’essai définitif visant à tester l’efficacité des voies d’administration des traitements de prévention d’IRA après les tomodensitométries avec agent de contraste.

## Background

Since its invention, computed tomography (CT) has greatly improved diagnostics and patient care. Over 50% CT procedures employ iodinated contrast material (CM) [1]. At the same time as the CT scan use has rapidly increased [2], it has been estimated that more than 80 million doses of CM are administrated worldwide each year [3]. Thus, since the number of CT scan examinations increase, their adverse effects, including renal failure, also potentially increase.

Acute kidney injury is a common form of nephropathy induced by contrast medium reported by several studies [4-10]. Contrast-induced acute kidney injury (CI-AKI) is associated with long- term adverse outcomes including stroke, myocardial infarction, long term dialysis dependence, and death [11-15]. The average in-hospital cost of CI-AKI is estimated at $10,345 with the 1- year cost of treating a patient with CI-AKI at $11,812 [16]. Thus, there is considerable interest in developing strategies aimed at reducing the risk of CI-AKI.

Hydration is clearly beneficial in prevention of CI-AKI [17]. The Canadian guideline for chronic kidney disease patients who have contrast-enhanced computed tomography scans is to give intravenous (IV) saline both before and after their CT scan. Recommended protocols require that saline be given in the one hour before and in the six hours following contrast administration and have been formulated for arterial contrast (e. g. cardiac or peripheral angiography), where patients do need a short stay for monitoring [18,19]. For contrast CT, this requires additional nursing time and resources to monitor patients during the infusion (approximately 7–8 hours per patient). This regimen requires significant health care resources as it requires a same-day hospital stay, nursing time as well as patient inconvenience.

Oral salt and water solutions are well tolerated and effective in prevention and treatment of dehydration [20-23]. Mechanistic studies have also established that oral sodium and water loading results in rapid (within 20 minutes) changes in physiology with a significant increase in cardiac output and subsequent diuresis [24-26]. We performed a systematic review of randomized trials that compared oral versus intravenous volume expansion for the prevention of radiocontrast-induced AKI [27]. Our search identified six small studies (average sample size n = 85), all done in patients receiving arterial contrast [28-33]. We pooled all six trials together in a meta-analysis and found no significant difference in the odds of AKI between the oral and intravenous fluid arms (summary odds ratio 1.2; 95% CI 0.46 to 3.10; P = 0.73). Taken together, these data show that there is no clear trial evidence favouring intravenous over oral fluid expansion. Moreover, mechanistic evidence suggests that a sufficient proportion of an acute oral salt and water load is absorbed to have measurable physiological effects within 30 minutes [24,34].

However, most of the trials for contrast nephropathy prevention have been in the setting of arterial contrast such as with cardiac catheterization, and not with venous contrast, such as computed tomography. The advantages of an oral route of volume expansion in this setting would include decreased use of hospital resources and cost, reduced time in hospital as well as improved patient comfort and convenience [33,35,36]. The 2012 guidelines from the Kidney Disease: Improving Global Outcomes (KDIGO) workgroup state that, “if confirmed in larger studies, the oral strategy could offer an equivalent and more practical approach in preventing a decline in renal function after contrast exposure, without accruing additional delay in hospital days or in-hospital mortality” [37]. In addition, the trials we identified in our systematic review were all small, none was powered to study important clinical outcomes.

We are conducting a pilot randomized trial with the objective to measure the feasibility of a trial (recruitment, adherence to intervention, completeness of follow up) and the safety outcomes (adverse effects with the intervention). This will provide crucial information required to facilitate the planning of an adequately powered, definitive, randomized controlled trial comparing the effect of oral salt and water administration versus intravenous saline in the prevention of CI-AKI occurring after an intravenous (IV) contrast administration. This protocol describes the design and methodology of the pilot randomized trial.

## Methods

### Design and setting

The study is designed as a single-centre, parallel-arm, randomized controlled pilot trial. Participants will be recruited from the Ottawa Hospital, an academic centre serving a catchment area of 1.3 million residents in Eastern Ontario (Ottawa and environs), Canada.

### Participants

Participants will be identified within the medical imaging department. Eligible patients are outpatients who are scheduled for a contrast-enhanced CT scan of the chest or abdomen. We plan to enrol 50 patients in this pilot trial. Since this is a pilot trial to assess feasibility, we did not carry out a formal sample size calculation. The rate of recruitment in this trial will allow us to plan the recruitment strategy and duration of the definitive trial. For the large definitive trial (planned as a non-inferiority trial) our preliminary estimates for sample size suggests we need to recruit 1030 patients.

### Criteria

#### Inclusion criteria

- Chronic kidney disease (as defined by a glomerular filtration rate (GFR) < 30 mL/min/1.73 m^2^ using the Chronic Kidney Diseases (CKD)-EPI formula calculated on the day of screening [38];
- Undergoing an outpatient intravenous contrast-enhanced CT of the chest or abdomen;
- Age ≥ 18 years

### Exclusion criteria

- Inability to give informed consent;
- Previously enrolled in this study;
- Any contrast-enhanced test in previous 14 days (to exclude patients who might have ongoing AKI from previous contrast exposure);
- Congestive heart failure defined as NYHA class III or worse [39];
- Uncontrolled hypertension defined as SBP greater than 180 mm Hg or DBP greater than 110 mm Hg at screening;
- Currently receiving dialysis treatments.
- The physician ordering the CT scan has specifically ordered intravenous saline, sodium bicarbonate or n-acetyl cysteine.
- Scheduled to receive oral contrast solution as part of CT scan procedure

### Patient identification and recruitment

A chart review will be performed on all patients with scheduled contrast-CT scans of the chest or abdomen by department personnel. Potential participants will be approached by phone calls as per local study-specific Research Ethical Board -approved procedures and Standard Operating

**Figure 1:**
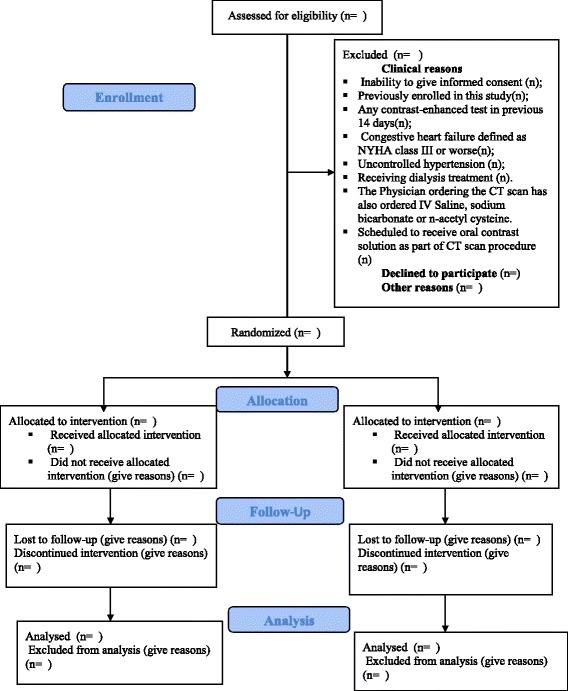
**Study flow chart.**

Procedures. Potential participants who consent will be invited for a screening visit where they will be asked to give a blood sample to analyze their serum creatinine (SCr) levels. This will serve to calculate the estimated GFR used in the screening process. Final inclusion/exclusion criteria will be assessed and confirmed by the Principal Investigator and Co-Investigator only. Consenting patients fulfilling entry criteria will be successively recruited. The Consolidated Standards of Reporting Trials flow diagram will be used to show the study flow chart (Figure 1.)

### Randomization and allocation concealment

Patients will be randomized to receive either oral salt and water or intravenous saline. The randomization process will consist of a computer generated random listing of the treatment allocations in permuted blocks of varying size. For example, given a block size of 4 patients recruited, there will be 2 allocations to oral salt and water arm and 2 to the intravenous saline arm. The system will have backup in the form of an independent statistician who will be informed and able to issue the treatment allocation in the event of a system malfunction/shutdown.

The randomization will be performed independently by a statistician after eligibility is documented by the Principal Investigator/Co-Investigators based on inclusion/exclusion criteria and after the patient signs the informed consent. From the computer random listing a code will be assigned to each new subject. This randomization code is a participant unique identifier (ID) and corresponds to the study treatment that will be given to the patient. Patient will be considered randomized as soon as the randomization code is given. All patients who are allocated a randomization code (i.e., study treatment number) will be irrevocably in the study and they will be followed until the end of study, loss to follow-up or death whichever comes first. The randomization codes corresponding to the participant ID will be printed and put in numbered sealed envelopes. On the day of the CT scan, these envelopes will be delivered to the imaging room to be opened by the Principal Investigator who will administer the trial treatment.

### Blinding

Blinding of the intervention is extremely difficult as one intervention will be oral and the other intravenous. A double-dummy design could be considered but the “dummy” intravenous fluid would likely have some volume expanding effect that could affect the outcome. Aside from the ethical consideration, a sham infusion could be considered but may prove difficult with back flow of blood from the venous cannula if no infusion were occurring. To minimize co- interventions that might be given once treatment assignments are known, participants, radiology staff and the ordering physician will not be informed of the treatment assignment prior to the scheduled CT, which will minimize the chance of the treating team providing additional therapies based on knowledge of the treatment assignment. All trial interventions will be administered by the Principal Investigator out of sight of imaging staff.

### Trial intervention and dosing

#### The oral salt and water arm

Participants randomized to this arm of the trial will receive 0.1 g/kg of salt (NaCl) in capsule form and 12 mL/kg of water. Patients weighing more than 110 kg will receive the same amount as per a 110 kg patient [18]. This dosage was based on the trial of Dussol et al. in which oral salt was given using a pre-specified, weight-based approach [32]. This trial showed that 50% more oral salt ingestion, compared to intravenous saline, produced equivalent urinary sodium excretion. We have used these proportions to formulate the equivalent oral salt dosing. The oral water dosing has been calculated to make the overall composition approximately isotonic similar to the intravenous saline arm. One third of the oral salt and water will be given in 1 hour before the CT and 2/3 in the 2 hours post-CT. Specifically, 0.03 g/kg of NaCl and 4 mL/kg of water in the one hour before CT and 0.07 g/kg of NaCl and 8 mL/kg of water taken over 2 hours post-CT. The salt capsule and water will be taken in presence of the research personnel who will record on an assessment form that the patient has received the intervention (ingested the oral salt and water).

### The intravenous saline arm

Patients randomized to this arm will receive IV isotonic (0.9%) saline which is the standard of care recommended by Canadian [19] and international guidelines [8,40-44]. The rate of isotonic saline will be 3 mL/kg given in the one hour before CT and 1 mL/kg/hour for 6 hours post-CT as per Canadian guidelines [19]. Patients weighing more than 110 kg will receive the rate as per a 110 kg patient [18]. The dose will be rounded up to the nearest 5 mL.

### Data collection

**Figure 2:**
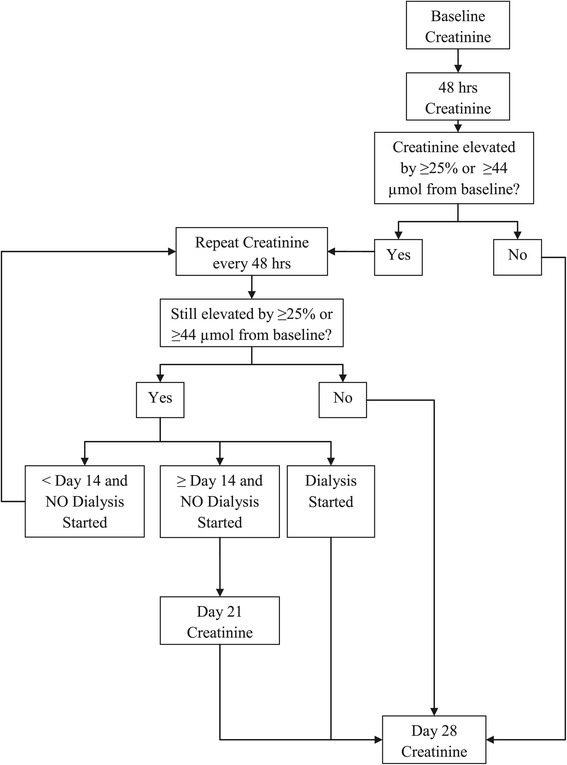
**Repeat creatinine test schedule.**

At baseline, participant demographic information (including ethnicity, sex, month and year of birth), CT scan characteristics, medical history and medication use will be recorded. On the day of the CT scan, before oral or IV fluids are given, a SCr will be measured that will be considered as the baseline value. A subsequent SCr level will be measured at 48 hours post-CT. If the SCr at 48 hours has increased by ≥25% or ≥44 μmol above the baseline value, the SCr will be repeated every 48 hours until it returns to within 25% of baseline or < 44 μmol above baseline or the patient needs to initiate acute dialysis. If the SCr has not returned to within 25% of baseline or < 44 μmol above baseline by day 14 post-CT, the SCr will be measured again on day 21. All participants (regardless of whether they developed AKI) will have SCr repeated on day 28 post- CT (Figure 2). The SCr will be measured in the biochemistry lab at the Ottawa Hospital. The repeat creatinine measurements, though underpowered for any assessment of efficacy, will allow us to ascertain the proportion of patients who follow-up at each time point, which will be critical for our larger definitive trial. The study timeline is shown in Table 1.

**Table 1:** **Study timeline**

	**Study period**							
	**Enrolment**	**Allocation**	**Post allocation**					**Close-out**
	**-t**_**1**_	**o**	**Day**_**1**_	**Day**_**2**_	**Day**_**3**_	**Day**_**4**_	**etc.**	**Day**_**28**_
Enrolment:								
Eligibility screen	X							
Informed consent	X							
Demographic data collection	X							
Medication use data collection	X							
serum creatinine testing	X				X			X
Allocation		X						
Interventions:								
Intravenous saline solution			X					
Oral salt water			X					
Assessments:								
Adherence to intervention			X					X
Adverse events			X					
serum creatinine testing					X			X

### Outcomes

Feasibility of conducting the definitive trial is the primary outcome for this pilot study. It will be determined by the proportion of patients who will 1) consent to enrolment in the study (recruitment rate), 2) complete the fluid ingestion at dose required in both arms (adherence to intervention), and 3) complete repeat measures of creatinine at 48 hours and 28 days (follow-up). Additionally, we will identify logistical issues related to screening, protocol implementation, randomization implementation strategy, time, and budget problems that can occur during the definitive trial.

The secondary outcome for this pilot trial is safety. It will be assessed by adverse effects occurring after receiving the trial treatment. Adverse effects that may occur because of the ingestion of water and salt include: nausea or gastrointestinal symptoms. Symptoms that may occur because of the saline solution or the technique of administration include discomfort with the venipuncture at the site of injection such as skin irritation and pain, hemorrhage, hematoma, thrombosis, phlebitis. All adverse events effects will be assessed by patients self-reported symptoms using a questionnaire with these symptoms listed and will be recorded in a Case Report Form at the end of the volume expansion protocol.

### Ethical issues and trial registration

The pilot trial will be conducted in accordance with Health Canada’s Good Clinical Practice guidelines [45] in accordance with the current Declaration of Helsinki and the Tri-Council Policy Statement: Ethical Conduct for Research Involving Humans [46]. All patients will be informed that they can withdraw from the study at any time. Patients who agree to participate to the study will provide a written informed consent. The study protocol and informed consent forms have been approved by the Ottawa Health Science Network Research Ethics Board. The trial is registered at the US National Institutes of Health (ClinicalTrials.gov) # NCT02084771.

### Statistical analyses

#### Baseline analyses

Baseline characteristics of patients in the two treatment arms will be assessed by calculation of frequency distributions and univariate descriptive statistics including measures of central tendency and dispersion.

#### Feasibility outcomes

For the recruitment rate, we will calculate the proportion (and 95% confidence interval) of all CKD stage 4 patients who are randomized into the trial among those who undergo a contrast-enhanced CT scan of the abdomen or chest. In addition, we will calculate the proportion of patients that were eligible but who declined consent, those who were eligible but were not approached. For the feasibility outcome of adherence, we will calculate the proportion of patients who will complete the fluid ingestion in both arms out of all those recruited. The follow- up will be measured by calculating the proportion of participant recruited who complete the follow-up (the proportion of patients who will undergo repeat measures of creatinine at 48 hours and 28 days among those recruited). Proportion of patients who will be adherent and those who will complete the follow-up will be compared between the arms with an unadjusted chi-square, or with Fisher’s exact test if the cell sizes are small.

#### Safety

For the safety outcome, we will calculate the frequency of overall adverse events that will occur in each trial arm and we will compare them using an unadjusted chi-square test or Fisher’s exact test. Univariate descriptive statistics including measures of central tendency and dispersion will be used to report the frequency of adverse events. This outcome will be underpowered to draw any definite conclusions, but will be conducted to help us design the definitive trial.

### Study management and patient safety

A trial management group involving the Principal investigator (SH), two Co-investigators (AA, GK) and study coordinator (EM) will review, implement and supervise all aspects of this pilot trial. The Data Management Services group of the Ottawa Hospital Research Institute will generate the random listing. Under the guidance of the Principal investigator and the study coordinator, the coordinating centre located at the Clinical Epidemiology Program of the Ottawa Hospital Research Institute will be responsible for receiving, processing, editing storing and analyzing all data.

A DSMB will have responsibility for the monitoring of adverse events throughout the study and will work independently from the trial. The DSMB will consist of three individuals with expertise in clinical trials, biostatistics, nephrology and radiology. All serious adverse events will be reported to and reviewed by the DSMB. The DSMB will recommend termination or continuation of the study in the event of an unexpected adverse event rate.

All investigational products supplies in the study will be stored in a secure, safe place, under the responsibility of the Investigator.

## Discussion

The proposed project is a pilot trial of a simple pragmatic intervention (oral salt and water) in comparison to the standard of care (intravenous saline) for prevention of acute kidney injury after IV contrast administration. The results of our pilot study will provide crucial data to the planning of a large multicentre randomized trial to determine if oral salt and water administration is as effective as intravenous saline in the prevention of radiocontrast-induced AKI occurring after an IV contrast administration. For this definitive trial, we will study efficacy (incidence of AKI) as primary outcomes, with secondary outcomes including clinical (hospitalization, need for dialysis, and mortality) and health services aspects (economic analysis). A lower recruitment rate in the pilot trial will help us to plan adding more sites or a longer recruitment period for the definitive trial. Protocol adherence and follow-up measurements will provide additional feasibility information to plan refinement of the final protocol as well as adjust sample size estimates for a larger trial. Knowledge of the frequency and severity of the adverse events will be used by the trial management group to determine if the potential benefits of conducting a larger trial outweigh the potential risks with the proposed intervention in a larger trial.

## Trial status

This pilot trial was designed in 2012–2013 and received a grant from the Department of Medicine of the University of Ottawa. Recruitment started in October 2014.

## Abbreviations

CT: Computed Tomography
CM: Contrast Material
CI-AKI: Contrast-induced acute kidney injury
KDIGO: The Kidney Disease: Improving Global Outcomes
CI: Confidence interval
GFR: Glomerular Filtration Rate
CKD-EPI: Chonic Kidney disease
NYHA: New York Heart Association
SBP: Systolic Blood Pressure
DBP: Diastolic Blood Pressure
IV: Intravenous
REB: Research Ethical Board
SOP’s: Standard Operating Procedures
SCr: serum creatinine
ID: Identifier
DSMB: Data safety and monitoring board
NaCl: Sodium Chloride
