# Erratum: Oral salt and water versus intravenous saline for the prevention of acute kidney injury following contrast-enhanced computed tomography: study protocol for a pilot randomized trial Salmonella blood stream infections in a tertiary care setting in Ghana

**DOI:** 10.1186/s40697-015-0064-7

**Published:** 2015-09-03

**Authors:** Swapnil Hiremath, Greg A. Knoll, Jeanne Françoise Kayibanda, Dean Fergusson, Benjamin J. W. Chow, Wael Shabana, Erin Murphy, Tim Ramsay, Matthew James, Christine A. White, Amit Garg, Ron Wald, Jeffrey Hoch, Ayub Akbari

**Affiliations:** Division of Nephrology, Faculty of Medicine, University of Ottawa, Ottawa, Canada; Clinical Epidemiology Program, Ottawa Hospital Research Institute, Ottawa, Canada; Kidney Research Centre, Ottawa Health Research Institute, Ottawa, Canada; Division of Cardiology, University of Ottawa Heart Institute, Ottawa, Canada; Department of Medical Imaging, Faculty of Medicine, University of Ottawa, Ottawa, Canada; Ottawa Health Research Institute, Ottawa Hospital, Ottawa, Canada; Faculty of Medicine, Epidemiology& Community Medicine, University of Ottawa, Ottawa, Canada; Departments of Medicine and Community Health Sciences, University of Calgary, Alberta, Canada; Division of Nephrology, Department of Medicine, Queen’s University, Kingston, Canada; Division of Nephrology, Department of Medicine, University of Western Ontario, London, Canada; Division of Nephrology, Department of Medicine, St. Michael’s Hospital and University of Toronto, Toronto, Canada; Institute of Health Policy, Management and Evaluation, University of Toronto, Toronto, Canada; Division of Nephrology, The Ottawa Hospital, Riverside Campus, 1967 Riverside Drive, Ottawa, K1H 7 W9 Ontario Canada

Following publication of [1] it has come to our attention that there was an error in the first author’s name which should be Swapnil Hiremath and not Hiremath Swapnil.
